# 

**Published:** 1858-05

**Authors:** 


### CONTENTS.

ORIGINAL COMMUNICATIONS— PAGE.

Case of Traumatic Occlusion of the Vagina. By Ernst Schmidt, M. D., 197
Case of Cancer of the Pylorus. By Ralph N. Isham, M. D........ ........ 204
Milk-Sickness. By J. P. DeBruler, M. D.. ........................ 206
Marshall Hall's Ready Method in Asphyxia of the Newly-Born, etc.; Hysteria from Chloroform. By J. W. Benson, M.D...................... 210
Two Cases of Ovarian Dropsy Successfully Treated. By J. H. Brower, M. D........................ 212
Case of Hæmoptysis. By Jas. M. Kindall.................................. 214
Case of Malformation. By S. W. Wallace, M.D........................... 215

Translations by Thomas Bevan, M. D.—

Researches on the Origin and Conditions of Existence of Eliptiform Convulsions, following Hemorrhage, and on Epilepsy in general. By A. Kussmaul and A. Tenner.... ................................ 216
Researches on the Influence of the Nerves in Inflammation. By H. Snellen........ ........ ....................................... 224

BOOK AND PAMPHLET NOTICES—

On the Use of Iron. By Isaac Casselberry, M.D............................ 225
An Essay on Prolapse of the Funis. By J. Gaillard Taylor, M.D......... 227
Lectures on the Sulphate of Quinia. By A. B. Palmer, M.D............... 229
Dysentery, its Pathology and Treatment. By R. Campbell, A.M., M.D. 229

EDITORIAL—

American Medical Association.............................................. 230
Illinois State Medical Society.............................................. 240
London Lancet.............................................. 240
Our Janitor.............................................. 240

